# 3D Genome Organization as an Epigenetic Determinant of Transcription Regulation in T Cells

**DOI:** 10.3389/fimmu.2022.921375

**Published:** 2022-06-22

**Authors:** George Papadogkonas, Dionysios-Alexandros Papamatheakis, Charalampos Spilianakis

**Affiliations:** ^1^ Department of Biology, University of Crete, Heraklion, Greece; ^2^ Institute of Molecular Biology and Biotechnology, Foundation for Research and Technology Hellas, Heraklion, Greece

**Keywords:** adaptive immunity, genome organization, epigenetics, thymocyte development, SATB1, autoimmunity

## Abstract

In the heart of innate and adaptive immunity lies the proper spatiotemporal development of several immune cell lineages. Multiple studies have highlighted the necessity of epigenetic and transcriptional regulation in cell lineage specification. This mode of regulation is mediated by transcription factors and chromatin remodelers, controlling developmentally essential gene sets. The core of transcription and epigenetic regulation is formulated by different epigenetic modifications determining gene expression. Apart from “classic” epigenetic modifications, 3D chromatin architecture is also purported to exert fundamental roles in gene regulation. Chromatin conformation both facilitates cell-specific factor binding at specified regions and is in turn modified as such, acting synergistically. The interplay between global and tissue-specific protein factors dictates the epigenetic landscape of T and innate lymphoid cell (ILC) lineages. The expression of global genome organizers such as CTCF, YY1, and the cohesin complexes, closely cooperate with tissue-specific factors to exert cell type-specific gene regulation. Special AT-rich binding protein 1 (SATB1) is an important tissue-specific genome organizer and regulator controlling both long- and short-range chromatin interactions. Recent indications point to SATB1’s cooperation with the aforementioned factors, linking global to tissue-specific gene regulation. Changes in 3D genome organization are of vital importance for proper cell development and function, while disruption of this mechanism can lead to severe immuno-developmental defects. Newly emerging data have inextricably linked chromatin architecture deregulation to tissue-specific pathophysiological phenotypes. The combination of these findings may shed light on the mechanisms behind pathological conditions.

## Introduction

The thymus and the bone marrow are the two primary lymphoid organs. In the former, thymus-specific lymphocytes (thymocytes), mature into T cells. T cells are fundamental for the maintenance of immune responses, homeostasis, and memory and are inextricably linked to mammalian survival, *via* modulating the adaptive immune response (1). In early embryonic development, the thymus is populated by hemopoietic stem cells that migrate from the bone marrow and home in on the thymus in two distinct waves. Before embryonic day 15.5 (E15.5), early thymic progenitors (ETPs) migrate and settle into the thymic tissue. ETPs are evolved to rapidly produce mature αβ T cells and innate γδ T cells. Upon this point, the second wave of colonization involves the lymphomyeloid-primed progenitors (LMPPs), which retain their potential to induce B and myeloid cell lineage specification (2–4).. When considering T cell development in the thymus, multiple cell types are expressed at various thymic regions that will then give rise to both effector (cytotoxic T-lymphocytes, Th1, Th2, Treg, Th17, Tfh) and memory T cells. If one were to assess the vast diversity of both effector and memory T cells, both CD4+ and CD8+ lineages play a fundamental role in regulating immune responses. Analyzing their epigenetic and transcriptional profiles provides essential information towards understanding the functions of these immune cells circulating the lymphatic system (5–8). T cell lineage specification kick starts from committed T-cell precursors called double-negative cells (DN) where no CD4 or CD8 glycoproteins are expressed. DN thymocytes can further be subdivided into four sequential developmental stages, distinguished by their surface expression of CD44, CD25, and CD3. The DN1 (CD44^+^CD25^-^CD3^-^) and DN2 (CD44^+^CD25^+^CD3^-^) cell types are found in the cortex of the thymus. At the DN3 (CD44^-^ CD25^+^ CD3Lo) stage TCRβ rearrangements occur, while at the DN4 (CD44^-^ CD25^-^ CD3Lo) stage, cells further develop into double-positive (DP: CD4^+^ CD8^+^) thymocytes following pre-TCR signalling. Both DN3 and DN4 cells are found in the subcortical thymic region, while the DP cells are located in the corticomedullary region (9, 10). The DP stage is the most essential in T cell development as TCRα rearrangements along with both positive and negative selection occur. Utilizing these multiple sorting processes, cells can then be distinguished according to their selection of CD4^+^ or CD8^+^ single-positive lineage (9–11). This procedure occurs in the medullary thymic region and the selected T cells exit the thymus and circulate through the lymphatic system (9–13). All these cellular processes and selections aim to assign proper T cell characteristics and avoid self- reactivity. They are truly intricate mechanisms and are governed by a plurality of factors as research into the topic has demonstrated thus far.

Epigenetic modifications consisting of DNA methylation (14), histone modifications (15), lncRNA function (16) and chromatin remodeling have been long known to play crucial roles in regulating gene expression and concomitantly in development, physiology and disease.

### DNA Methylation

Cytosine methylation is the most widely studied epigenetic modification with great implications in cancer development and genetic disorders (17, 18). DNA methylation is mostly detected in promoter regions and especially in CpG islands, which are clustered regions characterized from high G/C content (19). DNA methylation is a very dynamic process that varies according to the developmental stage and its perturbation is correlated with many genetic and epigenetic diseases (20). The vast majority of CpG islands consist of 5-methyl cytosine (5mC) (14). In mammalian genomes the Ten Eleven Translocation (TET) family proteins play a vital role in the hydroxylation of 5mC to 5-hydroxymethylcytosine (hmC) (21). Previous studies have highlighted that hmC is resistant to methylation from DNMT1, leading to DNA damage and depicting the importance of the global DNA methylation pattern in genome integrity (22). The binding pattern of TET1 in the genome intersects with CpG islands mainly of bivalent promoters (where hmC has been found to be highly deposited) and active promoters. Additionally TET family members interact with major complexes such as the Polycomb Repressive Complex 2 (PRC2) implicating their roles in gene regulation during development (23). CpG methylation mainly occurs in the promoter regions of genes in order to repress gene expression by either recruiting chromatin remodelers that will reorder the local chromatin structure (24) or in some cases by inhibiting the binding of transcription factors (25). On the other hand, apart from the inhibitory role of DNA methylation in binding, the DNA binding capacity of a big fraction of transcription factors, that play vital roles in major developmental processes are favored by 5mC deposition (26).

### Histone Modifications

Different histone modifications have been long known to play crucial roles in forming chromatin structure and regulating gene transcription (27, 28). The nucleosome is the fundamental element of chromatin and is composed of a histone octamer (two from each H2A, H2B, H3 and H4) wrapped around 146bp of DNA (29, 30). These fibers can be observed with an electron microscope as beads on a string structure which are separated by linker DNA. Such sub-nuclear organization favors the packaging of approximately two meters long DNA in each nucleus, but this is not the only reason of its existence. Interphase chromosome organization plays a crucial role in gene regulation in order to construct a specific transcription programme. The level of chromatin compaction determines whether specific transcription factors are able to bind chromatin and regulate gene expression (31–33). More specifically, covalent modifications in the exposed amino-terminal tails of histones in nucleosomes can change the compaction rate of chromatin. For example, histone acetyltransferases (HATs) such as CBP/p300, GCN5 and PCAF catalyze the acetylation of several amino acid residues to promote binding of transcription factors, utilizing their bromo-domains. Such an important histone modification is H3K27ac, that marks active enhancers (34). Acetylation is reversed by histone deacetylases (HDACs) which leads to transcriptional repression. Lysine methylation is catalyzed by histone methyltransferases (HMTs) such as Setd1a and Set2 that catalyze activatory histone modifications (H3K4me1/2/3) or G9a and Suv39H1/2 that catalyze repressive modifications (H3K9me). Methylated histones are recognized by protein complexes encompassing chromo-domains and plant homeodomains (PHD). Histone methylation is reversed by histone demethylases (KDMs). Therefore, several enzymes either by remodeling or stabilizing chromatin structure can affect RNA Pol II recruitment and finally regulate the transcriptional onset (35–37).

### Long Non-Coding RNAs

Non-coding RNAs are long known to mediate specific roles in gene expression through the blockade of mRNA translation (38, 39). In the recent years, lncRNAs are emerging as key regulators of the epigenome. A vast majority of lncRNAs are being produced and processed in a similar fashion as mRNA molecules, but in contrast lncRNAs have higher turnover and mainly reside in the nucleus. Additionally, due to the negative charge of RNA molecules, lncRNAs can neutralize the positive charge of histone tails in order to alter chromatin compaction, implying a potential role in transcriptional regulation (16, 40). Important chromatin organizers such as CCCTC-binding factor (CTCF) and Yin-Yan 1 (YY1) are known to interact with RNA species in order to co-excerpt their regulatory functions upon chromatin (41–44). One of the most well-known and studied lncRNA is Xist which plays a fundamental role in X chromosome inactivation (45). Recent work from the laboratory of Edith Heard, using allele specific HiC approaches, demonstrated that the inactive X chromosome consists of two mega domains and although very few TADs are present they harbor facultative escapee genes (46). The deregulation of X-chromosome inactivation from Xist can lead to severe autoimmunity (47–49), highlighting the vital role of Xist in safeguarding the X-chromosome genome organization and thus the physiology of several cell types. Various studies have indicated the production of non-coding transcripts from enhancer regions, termed enhancer long non-coding RNAs (elncRNAs) and enhancer RNAs (eRNAs) (50). Previous studies have identified eRNAs transcription as a crucial process for enhancer activation and transcriptional regulation which can be further supported with genome-wide analyses of Pol II occupancy and active transcription in many animal models (51–54). eRNAs are able to activate transcription by recruiting transcription factors and coactivators altering chromatin conformation of specific genomic regions (55–57). It is speculated that enhancer transcription preceeds gene expression in a manner that helps stabilize an open chromatin environment for transcription factor recruitment and maintenance of transcription onset (58). As lncRNAs are shown to exert gene regulatory roles, enhancer transcription and production of specific lncRNAs from specific genomic regions suggests their implication in tissue/cell specific epigenetic and transcriptional regulation.

### T Cell-specific Transcription Regulators

T cell development is based upon complex signaling pathways and expression programs that are unique to each cell lineage, including hematopoietic progenitors up to single positive T cells (59, 60). Aside from the aforementioned transcription factors (CPB/p300, GCN5, PCAF), tissue specific factors also play crucial roles in the development, commitment and differentiation of T cell lineages. The activation of the Notch signaling pathway, in the beginning of thymic development, leads to the upregulated expression of crucial transcription factors such as TCF1 and GATA3, both indispensable for the proper commitment of the early T cell progenitors (61–63). Specifically, TCF1 acts at the stage of ETP cells and binds to regulatory regions that will become accessible at later developmental stages, in order to shape and pre-pattern the genomic landscape of committing T cell progenitors (61). Accordingly, GATA3 serves a major role in early T cell commitment, acting in concert with the Notch signaling pathway in order to repress the expression of genes that would promote the B cell fate (64–66). Additionally, GATA3 is essential at the DP stage for promoting the CD4 T cell lineage fate over the CD8 fate (67, 68). Another group of pioneer factors necessary for proper T cell development is the Ikaros family, whose role is vital for checkpoint licensing and supporting the fast transitions between expression programs necessary to each stage (69). Of equal importance are members of the RUNX family of factors, with members such as RUNX1, whose role is vital at the DN stage and RUNX3 which is a pioneer factor for CD8 cell lineage specification, by silencing the CD4 cell lineage (70, 71). Finally, one of the most important transcription factors that regulate T-cell commitment is Bcl11β, whose expression starts at the DN2a stage and plays an important role in the transition from the DN to the DP stage, as well as in the β selection of T cells (72–74) and controls the balanced temporal specification of CD4 and CD8 T cell lineages (75). The role of Bcl11β is discussed in greater detail in the next chapter as it functions by mediating changes in the 3D chromatin organization of specific gene loci, supporting 3D chromatin interactions that are crucial for the DN to DP transition (76).

Although the aforementioned epigenetic mechanisms and transcription factors are more extensively studied, novel mechanisms and players, such 3D genome organization and chromatin organizers, are gaining great interest the last few years.

## 3D Chromatin Organization as an Epigenetic Determinant of T Cell Development

### General Principles of 3D Genome Organization

**Figure 1:**
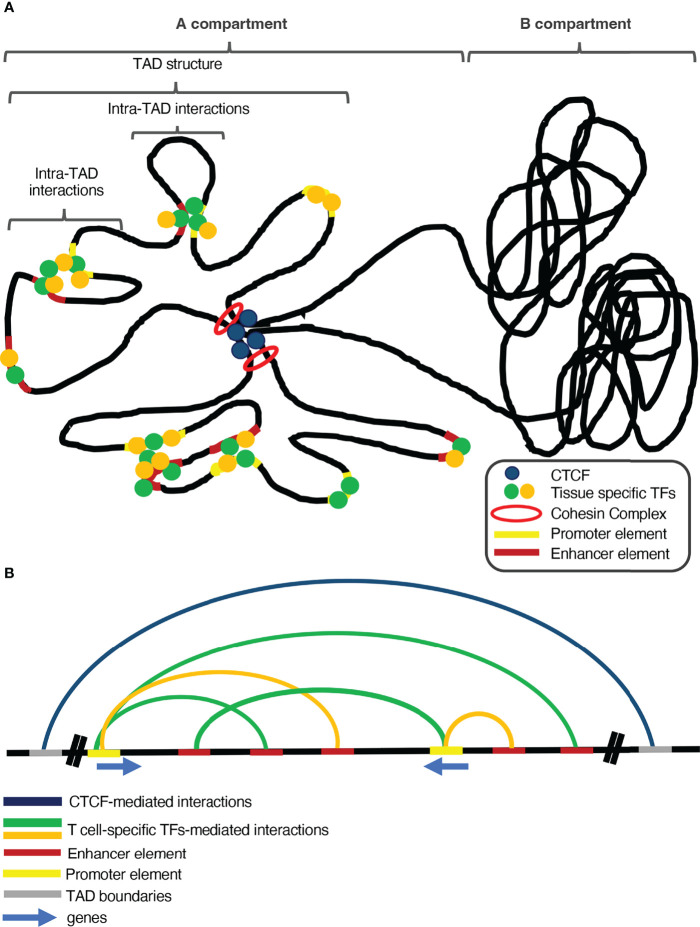
The interplay between global and tissue-specific genome organizers and transcription factors is vital for tissue-specific gene regulation. **(A)** Global genome organizers (such as CTCF and the Cohesin Complex) safeguard the genomic integrity and shape the general structure of the genome, by insulating the Topologically Associating Domains (TADs) from each other. Additionally, loop extrusion within TADs facilitates long-range chromatin interactions between regulatory elements and genes. Under the control of the aforementioned global organizers and inside such sub-structures of the genome, tissue-specific transcription factors and chromatin remodeling complexes control proper spatiotemporal gene expression, during development and differentiation. **(B)** Global genome organizers cooperate with tissue-specific transcription factors and chromatin organizers to sustain tissue-specific transcription programs.

In the last few years, research in the field of 3D genome organization has grown tremendously, specifically in favor of exploring the role of chromatin conformation in determining transcription programs. The chromatin fiber in a cell resembles the structure of a polymer due to its long and repeating territories and compartments, which helps a lot to understand its dynamics and genomic interactions. If two loci are in close proximity, there is a higher probability for them to physically interact, especially with the help of proteins, although such gene specific interactions are difficult to happen by chance in the eukaryotic cell nucleus. Therefore, due to the low probability that two randomly placed genomic loci would interact, the genome has to undergo highly dynamic conformation changes, in order to reshape accordingly the gene expression patterns (77). Mammalian genomes are densely packed and organized inside the cell nucleus in a very sophisticated manner in order to favor precise regulation of gene expression (78). The different districts of the genome are segregated into distinct self-interacting domains, called topologically associating domains (TADs), which play a significant role in local gene transcription regulation (79). These TADs cluster together according to their chromatin conformation status, in A and B compartments, with A being regions of more open chromatin structure and near the interior of the nucleus, whereas B being more compact and localized near the nuclear lamina (80). This compartmentalization can be explained by many diverse mechanisms such as i) the polymeric nature of the chromosomes that favors the interactions of proximal elements, which drives the clustering of each type of compartment together and separated from the other type (81), and ii) microphase separation (82), which is based on weak interactions between chromatin regions that share the same structural and/or functional characteristics forming separated condensates. Additionally, the pairing of sister chromatids, the transcriptional onset and DNA affinity can also be considered as potential mechanisms of chromosomal compartmentalization (83). Genome organization is crucial in regulating gene expression patterns and in many cases the capacity of TADs in insulating specific genomic regions is important to restrain ectopic promoter-enhancer communications (84, 85) (**Figure 1**). The boundaries of many TADs in mammalian genomes are marked by the global chromatin remodeler CCCTC-binding factor (CTCF) (86), which safeguards their integrity, since depletion of CTCF leads to massive disruption of these subchromosomal territories (87–89).However, there are several TAD regions whose structure is independent of CTCF or Cohesin binding and are probably formed *via* transcription-mediated enhancer-promoter communications which are regulated by more specialized or tissue-specific transcription factors (90).

CTCF binding in the boundaries of TADs is the main feature that differentiates them from the compartments, which are larger regions and control the general pattern of chromatin conformation (79, 91). The compartmentalization of chromosomes based upon CTCF is facilitated *via* loop extrusion and is mediated by the cohesin complex (92), a group of subunits that form a ring-shaped protein complex, which is loaded on the genome and facilitates the extrusion of chromatin loops up to the point where it reaches a CTCF-bound site (93, 94). Loss of the cohesin complex subunits leads to loss of CTCF-mediated loops, leading to great isolation between A and B compartments and chromatin de-compaction (95, 96).The formation of chromatin loops is not a permanent state, as loop extrusion is a very dynamic process which coincides with the gene expression program of the different developmental stages of quite diverse cell lineages (89).

### The Interplay Between 3D Genome Organization and Transcriptional Regulation

Loop extrusion facilitates the communication of enhancers with their cognate promoters and isolate them from foreign regulatory elements to block unwanted regulatory interactions (87, 97). In general, each TAD contains gene loci that present a coordinated transcriptional program compared to gene loci outside the TAD (98), highlighting the importance of sub-chromosomal organization in gene expression. Studies in *D. melanogaster* and mouse embryos have indicated that transcription initiation during the early developmental stages correlates well with specific chromatin compartments in the nucleus. Interestingly transcription inhibition in some cases leads to disruption of the chromosomal domains, indicating a possible link between genome organization and gene regulation (99–101). Additionally, genomic studies in macrophages infected by the Influenza A Virus (IAV) showed that upon infection, extensive genome reorganization mainly occurs in infection-related genes, which correlated positively with transcription elongation. Interestingly, the utilization of transcription inhibitors such as flavopiridol, decreased transcription accompanied by diminished chromatin compaction of the IFNβ-induced genes and increased cohesin occupancy and loop strength in the transcribed genes, reminiscent of chromatin compaction (102).

However, many studies depict no causative relationship between the two processes. Specifically, genomic studies utilizing Micro-C in combination with PolII ChIP-seq identified that although transcriptional inhibitors block transcription initiation, there are no changes in genome organization, neither in compartments nor TADs and loops. Additionally, the region of active transcription presented a more compact chromatin state and highly expressed genes formed more 3D chromatin interactions than low expressing genes and regions (90). Depletion of the Cohesin loading factor Nipbl (Δ*Nipbl*) led to diminished Cohesin loading onto chromatin and loss of TAD structures without major changes in A/B compartmentalization. As all gene regions equally lost their TAD organization independently of gene expression status and activating histone marks of regulatory elements (H3K27ac, H3K4me3) were unperturbed, the disorganization state in the Δ*Nipbl* cells did not depend on the altered transcription onset. On the other hand, although loss of Cohesin led to extensive deregulation of expression, these genes were not implicated in context-specific functions and seemed to act like a response to the loss of Cohesin loading (95). Blockade of DNA replication or transcription has been found to exert little effect on Cohesins’ loading whereas site mutations of Cohesins’ ATPases severely reduced Cohesins mobility to reach CTCF anchors. On the contrary, when deleting the CTCF motifs near highly expressed genes, loss of loop extrusion leads to diminished transcription (103). Pioneer work in the field, from the lab of Gerd Blobel, has elegantly demonstrated that forced chromatin looping in the regulatory elements of γ globin, *via* targeting of the self-association domain of Ldb1, can lead to reversion of its epigenetic silencing and reactivation of the gene (104).

### 3D Genome Organization of Developing T Cells Drives Tissue Specific Gene Expression

T cells undergo major changes in their gene expression programs as they develop in the thymus, a process which requires extensive 3D genome plasticity. DNase-seq experiments, utilizing T cells in different developmental stages, indicated a major reorganization of chromatin in genes that express pioneer factors, during the transition from CD4^-^CD8^-^ (double negative, DN) DN2 to DN3 and from DN4 to DP cells. The genes that exhibited increased chromatin accessibility in hematopoietic stem progenitor cells and DP cells were encoding for regulators of T cell lineage commitment. Analysis of TAD dynamics between the aforementioned transitions indicated positive correlation between them, revealing a cooperative reorganization throughout T cell development. Interestingly, increased intra-TAD interactions coincided with gene expression of adjacent genes and positively correlated with the upregulation of genes encoding for pioneer factors that drive differentiation and commitment of T cells with concomitant stable transition of B to A compartments (105).

Studies in CD4^+^CD8^+^ (double positive, DP) thymocytes have shown that *Rag* ^-/-^ deficient DP T cells present monoallelic recombination of *Tcra*. Only one of the two *Tcra* alleles colocalizes with γ-H2AX foci (indicative of double strand breaks) and looping away from the chromosome 14 territory, towards a more euchromatic region, favors transcriptional initiation in a RAG independent mechanism (106). Upon T cell receptor (TCR) stimulation, T cells undergo rapid and extensive chromatin reorganization, characterized by decondensation (euchromatinization), accompanied by increased size of the nucleus and the cytoplasm, indicative of an activated state of T cells (107–110). These changes in the nucleus are independent of any altered CpG methylation levels or differential histone modifications deposition. In the prior developmental stages, highly proliferative DN T cells present a larger size which due to the mode of action of Condensin II leads to the compact state of quiescent thymocytes. Loss of a condensin II subunit leads to disrupted T cell development due to delayed β selection, resulting to loose nuclear conformation and apoptosis (111). The ectopic opening of chromatin leads to the accessibility of STAT5 in genes destined to be expressed in later lineages and concomitant ectopic activation of peripheral T cells (111). These findings link the importance of the 3D genome organization in spatiotemporal gene regulation with the deregulated phenotype and expression observed in autoimmune diseases.

Genomic studies, in *ex vivo* TCR activated T cells, have revealed the markedly open chromatin state of transcription factor DNA binding sites and genes that are crucial for CD4^+^ T cell differentiation and activation. Additionally, there was positive correlation between this increased chromatin accessibility and genomic interaction strength with upregulation of gene expression. Importantly, gene loci harboring autoimmune disease-associated single nucleotide polymorphisms (SNPs) were moved towards accessible chromatin regions upon activation and had the ability to participate in long-range chromatin interactions with genes linked to Rheumatoid Arthritis progression (112). This study depicted the importance of 3D genomic studies in the search of new therapeutic targets for inflammation and autoimmune diseases.

DamID analysis of activated Jurkat T cells indicated that the vast majority of actively transcribing genes reside outside Lamin Associating Domains (LADs) of the cell nuclear periphery. Additionally, genes involved in cell cycle progression and early effector functions are highly depleted from LAD regions with concomitant high gene expression. Interestingly, upon activation of T cells, important cell lineage specification genes are actively repositioned away from the nuclear lamina, to the interior of the cell nucleus, in order to communicate with their enhancer elements (113).. These features, highlight once again the safeguarding role of the 3D genome in T cell specific gene regulation upon activation.

As mentioned before, the function of lncRNAs is an important epigenetic mechanism of transcriptional regulation. A revolutionary work from the lab of Cornelis Murre and colleagues showed that the intergenic regions of the *Bcl11b* gene locus, expressing the master transcriptional regulator Bcl11b, whose role in T cell commitment is vital, repositioned from B to A chromatin compartments and from the repressive nuclear lamina to the nuclear interior, specifically at the DN2 stage during thymocyte development. This enhancer region was found to produce a long non-coding RNA (lncRNA), namely ThymoD, whose expression positively correlated with this of *Bcl11b*. Surprisingly, loss of ThymoD lncRNA led to the decreased expression of *Bcl11b* in DN2 thymocytes, but not of the cognate genes in the nearby loci. In a very peculiar mechanism ThymoD transcription acts *in cis* and drives the detachment of *Bcl11b* locus control region from the nuclear lamina, by demethylating CpG islands in the region, in order for CTCF to bind and by recruiting the cohesin subunit Smc3, to facilitate repositioning and promoter-enhancer communication in the *Bcl11b* locus (76).

### Dis-Organization of 3D Architecture Results in Pathological Conditions

As 3D genome organization is more and more linked to gene regulation and tissue specific gene expression to safeguard proper development and physiology, an emerging aspect is the pathological consequences of 3D genome dis-organization. Genomic studies using Jurkat cells, identified that the vast majority of the known genes implicated in T-ALL are confined in insulated TADs. More specifically, a good fraction of recurrent deletions, known to drive proto-oncogene activation, are found to intersect with insulated TAD borders and have been previously identified in T-ALL. CTCF binds the borders of several TADs that engulf T-ALL associated genes and the deletion of these boundary CTCF sites led to the transcriptional up-regulation of these genes, indicating the crucial role of TADs structure in safeguarding cell physiology (114). A seminal work from the lab of Peter Fraser and colleagues developed and utilized promoter capture Hi-C (PCHi-C) in order to identify promoter interacting regions (PIRs) in 17 human blood cell types. Apart from the lineage specificity in chromosomal interactions and cell-type-specific enhancer-promoter interactions that were identified among these cell types, PIRs overlapped or were proximal to expression quantitative trait loci (eQTLs) disease-associated SNPs. Surprisingly, taking into consideration the 421 highest scoring genes as well as their putative interactors and pathways, they constructed a network of disease susceptible regions from which the vast majority (76%) were previously unknown (115). This study depicts the importance of 3D genome reorganization in cell and tissue-specific gene regulation during development and how its dis-organization can lead to the deregulation of disease-associated phenotypes. In a similar fashion, genomic studies from the laboratory of Howard Y. Chang utilized H3K27ac HiChIP in several human T cells to identify functional enhancers and their role in disease-associated DNA elements, based on enhancer interaction signals (EIS). In concordance to the previous study, the EIS contacts were cell-type specific. By comparing the EIS of the 3D data (HiChIP) to 1D data (ChIP-seq, ATAC-seq) they identified 36% of EIS that were specifically dependent on 3D genome interactions. By combining previously known PICs-SNPs in several T cell subsets with 1D and 3D data they demonstrated high EIS scores associating with autoimmune disease related SNPs with high cell specificity. These HiChIP-based EIS could reliably designate functional causal variants in haplotype blocks (116). These findings indicate that HiChIP 3D interaction data can be used in order to predict candidate target genes of disease-associated SNPs with high confidence in several T cell subsets and shed light in the deregulation of molecular mechanisms that lead to autoimmune phenotypes. Genomic studies using wild type and non-obese diabetic (NOD) mice revealed the increased accessibility of disease-related regions before the disease onset. Although only 133 highly interacting regions were identified bearing 1467 regulatory elements, they are controlling important immune-related genes, highlighting the role of the underlying 3D chromatin network in T cell development. Interestingly, the genes that are associated with these hyperconnected regions in NOD mice have prominent roles in T cell activation and some of them were previously implicated in autoimmune diseases. Additionally, the NOD-specific hyper-connected regions intersected with CTCF binding sites and encompassed CTCF-binding motifs in contrast to the WT mice. By backcrossing NOD with WT mice, to generate heterozygous F1 mice, decreased interaction was identified in disease-related regions indicating that dis-organization of 3D interactions was mediated in-cis *via* genomic variants that deregulate the function of enhancer networks (117).. *In situ* HiC performed in 8 T-ALL samples and cell lines as well as peripheral T cells indicated 3D genome interaction changes in concordance with expression changes from the WT state as well as between the ALL samples. More specifically, the widely studied *MYC* oncogene, which is upregulated in many T-ALL cases, resides in a TAD that presents increased inter-TAD interactions in T-ALL samples. Indeed, 4C-seq using the *MYC* promoter as a viewpoint revealed increased enhancer-promoter interactions in the T-ALL samples compared to the controls. Inhibition of NOTCH1 signalling, that activates many ALL-associated genes led to decreased enhancer activity and 3D interactions in NOTCH1-regulated regions but not in the *MYC* locus regulatory elements. Surprisingly, CDK7 which is insensitive to NOTCH1 inhibition, regulates the expression of *MYC* since treatment with a CDK7 inhibitor led to decreased 3D interactions and enhancer activity of this specific gene (118). This study demonstrated the complex regulatory patterns in different blood cancers and the targeted function of distinct protein players in shaping the 3D interactions of specific disease-associated genes.

## Global 3D Chromatin Organizers and Their Role in T Cell Specific Transcriptional Regulation

### CTCF

The master chromatin organizer CTCF is long known to play a pivotal role in safeguarding and controlling the integrity of the 3D genome (86). The first studies on CTCF that were conducted in the lab of G. Felsenfeld and colleagues, employed enhancer activity assays for the β-globin gene and identified that CTCF bound a specific regulatory element that served as an enhancer blocker, which corresponded to an insulation site in this TAD (119). Additional work has highlighted that CTCF was constitutively bound to a DNase I hypersensitive site at the 3’ of chicken β-globin locus, throughout several developmental stages (120). ChIP-loop assays have shown that CTCF interacted with an imprinting control element (ICE) of the *Igf2* locus, in order to regulate its expression (121). In a pioneer study from the laboratory of Bing Ren, chromatin immunoprecipitation combined with genome tilling arrays identified 13804 binding regions of CTCF in IMR90 (human embryonic lung fibroblasts) cells. Interestingly, CTCF was mainly enriched at intergenic regions, that did not overlap with known transcription factor binding sites and localized approximately 48kb away from gene promoters (122). Loss of CTCF leads to major disruption of TADs and their insulation from each other, but does not affect the overall genome architecture, as indicated by the insignificant changes in the structure of A/B compartments (123). An important study in CD4 Th1 cells depicted that CTCF plays a vital role in regulating the expression of effector genes in an IL-2 and α-ketoglutaric (αKG) signaling dependent fashion. Specifically, CTCF binding sites correlate with 40% of the genes induced *via* αKG. Moreover, knockdown of *Ctcf* decreased the expression of IL2 and αKG sensitive genes. Upon Il2 and αKG treatment the interaction strength within the CTCF mediated TADs was increased in the IL2/αKG sensitive genes (124). This study demonstrated the link between immune-related metabolic patterns with tissue specific 3D gene regulation programs. Conditional knockout animals that harbor deletion of *Ctcf*, at the DN2 stage of thymocyte development, display decreased cell populations accompanied by extremely low levels of DP and SP cells in the thymus and CD4^+^ and CD8^+^ SP cells in the secondary lymphoid organs. Interestingly, increased numbers of immature single positive (ISP) cells pointed out that CTCF is necessary for αβ T cell proliferation and growth, without affecting the proper rearrangement of their V(D)J fragments (125). Computational analysis, using digital DNase I profiling, identified a potential binding site of CTCF close to a DNase I hypersensitive region of the *Ifng* locus. ChIP experiments verified its increased binding in T helper type 1 (Th1) and Th2 cell lineages, in contrast to naïve CD4 T cells. Co-occupancy of CTCF and the master regulator of Th1 cell lineage, T-bet, was observed in Th1-specific CTCF binding sites and depletion of CTCF disrupted the 3D chromatin landscape of the *Ifng* locus, leading to its decreased expression, but without any major effect on T-bet’s DNA binding or expression. Alternatively, loss of Tbet led to a more dramatic decrease in *Infg* mRNA levels, depicting that a tissue-specific regulatory factor may guide a general chromatin organizer to be recruited on tissue-specific genes (126). The presence of a knocked in enhancer blocker (H19-ICR), which is effectively bound by CTCF near the TCRβ locus, did not dramatically affect T cell development in the thymus. Interestingly, the CTCF-dependent insulation lead to differential promoter-enhancer interactions and concomitantly alternative segment usage (127).. ChIP-seq experiments for CTCF have indicated its binding in the regulatory regions of many V gene segments in DP thymocytes and a high dependency on the presence of the Eα enhancer element, which regulates *Tcrα* expression (128). Indeed, conditional knockout of *Ctcf* at the DN stage of thymocyte development (*Ctcf* ^f/f^ *Lck*-Cre) indicated the problematic recombination between the Vα-Jα segments and the decreased chromatin communication between the Eα and T early alpha (TEA) promoter, that control *Tcra* expression, accompanied by decreased mRNA expression. Surprisingly, loss of CTCF increased the interaction of Eα with *Tcrd* gene segments, unravelling its important developmental role in safeguarding the proper development of αβ DP T cells (128). CTCF was also found to be recruited in asthma risk-related SNPs of the *ORMDL3* gene and increased the activity of its enhancer in naïve CD4 T cells, which was essential for the repression of *Il2* expression in the same cell type, a mechanism that may contribute to asthma progression (129). Loss of CTCF or its binding sites in the V_H_ region of the *IgH* locus in B cells disrupted the formation of three enhancer-independent chromatin loops in the region (130). Interestingly, studies of the *IgH* locus recombination in thymocytes, revealed that CTCF indeed binds in the same regions in DP T cells but fails to regulate the proper recombination events of the V_H_ segments. This finding highlights the importance of the interplay of CTCF with tissue specific regulators for proper chromatin conformation and expression (131). CTCF was found to cooperate with the transcription factor OCT1, in order to facilitate interchromosomal interactions between the *Th2* locus and the *Il17* locus. Importantly, depletion of CTCF led to abolished binding of OCT1 in the RHS6 DNase I hypersensitive site of the Th2 locus. Loss of any of these two factors led to diminished interchromosomal interactions between the two gene loci, in naïve CD4^+^ T cells and interestingly to the upregulation of IL17A production and the commitment of these cells towards the Th17 cell lineage. Therefore the synergistic function of tissue specific and global factors is vital for the regulation of the three dimensional organization in cytokine loci, important for determining alternate T cell lineages (132).

### The Cohesin Complex

Members of the cohesin complex were firstly discovered in studies that underlined their role in sister chromatid cohesion, chromosome condensation and segregation, during the progression of the cell cycle, as well as their role in safeguarding the integrity of the genome throughout the cell cycle in *D. Melanogaster* (133), *S. cerevisiae* (134–136) and *X. Laevis* (137). Cohesin subunits have been found to interact with specific regions near telomeres and centromeres in a kinetochore protein dependent fashion and only the regulatory elements that have been primarily activated seemed to associate with subunits of the cohesion complex, indicating a form of epigenetic memory through chromatin structure (138). Cohesin association with transcriptional regulation was initially described by studies in *S. cerevisiae* where promoter deletion of a highly expressed gene, led to disrupted cohesin binding in the region. Additionally, transcription initiation could alter the localization of cohesin towards or away from specific genomic regions and the binding pattern of RAD21 subunit highly correlated with regions of active transcription (139).

In mammalian cells, the Cohesin Complex has been extensively investigated during the past decade as a critical ally of CTCF, in supporting genome architecture. The Cohesin Complex consists of two Structural maintenance of chromosome (SMC) proteins (SMC1, SMC2) that form a ring-shaped structure and are interconnected at the upper end, shaping a hinge. The other side of the ring (ATPase heads) is held together by Scc1, a kleisin subunit that facilitates ATP dependent dimerization of the SMC subunits whereas the Scc3 subunit forms around the Scc1 and possibly plays a role in Cohesin loading (140). The major role of Cohesin in genome organization relies in its ability to form or maintain chromatin loops. Although many models have described Cohesin’s function, the loop-extrusion model has gained a lot of attention the recent years, as it proposes that Cohesin primarily targets a small loop and facilitates its enlargement by passing it through its ring while this region is being fend of the general structure of the domain (141).

In the pioneer study from the laboratory of Matthias Merkenschlager, it was shown that the binding of the cohesin complex on specific DNase I hypersensitive regions, of immune-related genes (*preb1/Igll1*, *Cd8*, and *Cd4*), was sequence specific. In contrast to yeast, mammalian cells display an even distribution of cohesin binding between genes and intergenic regions (142).

The Hadjur et al. study was carried out in CCR5^+^ Th1 cells and depicted that cohesin, alongside CTCF, binds in the boundary elements of the *Infg* gene and regulates its expression, by mediating promoter-enhancer interactions. Loss of either CTCF or cohesin led to decreased contact frequencies between the *Ifng* gene and its regulatory elements. However, only in the absence of RAD21, *Ifng* mRNA and protein levels were reduced, although the binding pattern of CTCF was not altered, depicting that only cohesin’s function was necessary and not CTCF’s in driving gene regulation and *Ifng* expression in CCR5^+^ T cells (142). ChIP experiments for cohesin subunits, in T cell lines, uncovered its ability to bind highly transcribed genes that undergo extensive locus reorganization and an overlapping binding pattern with CTCF. Depletion of CTCF extensively disrupted the association of cohesin with chromatin, indicating that CTCF could recruit the cohesin complex on DNA, to cooperatively facilitate TAD insulation (143). Depletion of cohesin, at the DP stage of thymocytes, did not affect the proliferation of the cells in the thymus, but led to apoptosis upon T cell activation. Additionally, loss of cohesin weakened the long-range interaction between Eα and TEA promoter in the *Tcra* locus. These findings indicate the synergistic role of cohesin and CTCF in regulating the insulation and proper recombination of *Tcra* locus segments, events that are crucial in T cell differentiation and development (144).

### Yin Yang 1

Yin Yang 1 (YY1) was primarily identified as a transcriptional regulator with contradictory functions. It was shown that YY1 actively binds the p5 promoter of the adenovirus early region 1 gene (*E1a*), responsible for expressing E1A proteins, and is shown to repress its expression, since the loss of either YY1 or its binding sites in these regulatory elements, ultimately restored E1A protein expression (145). Additional studies also revealed the activating role of YY1, as it could activate transcription of *E1a via* binding in the p5 promoter element in HeLa cells, while depletion of YY1 from the protein extracts, using a YY1 specific antibody, completely impaired transcription *in vitro* (145). YY1 was shown to physically interact with the transcriptional coactivator p300 and their interaction was important to guide E1A binding in the YY1-bound promoter, in order to alleviate the transcriptional repression of the p5 promoter (146).

Studies in Jurkat T cells have revealed that YY1 has multiple binding sites throughout the *Il4* promoter elements and specifically near the binding sites of NFAT. Silencing of YY1, deletion of its binding site (Y0) or mutation of its DNA binding domains led to major transcriptional repression of the *Il4* gene. YY1 was also able to induce promoter activity and increased IL4 production in primary T cells (147).

*In silico* analysis and electromobility shift assays utilizing the RHS7b element, of the Th2 locus, indicated YY1 as a potent binding factor and YY1 ChIP-seq experiments in Th1 and Th2 cells revealed its binding to several regulatory elements of the genes in the Th2 locus. The role of YY1 in Th2 cytokines production is vital, since ectopic expression of YY1, leads to their ectopic production whereas silencing of YY1 blocks their production. Quite importantly, loss of YY1 substantially reduced chromatin interactions between the *Il4* promoter and the promoter regions of *Il5*, *Il13* and *Rad50* genes, as well as its enhancer elements (148), highlighting the important role of YY1 in Th2 locus expression and Th2 cell development and activation.

A pioneer study from the laboratory of Richard A. Young and colleagues in mESCs utilized ChIP experiments for H3K27ac and H3K4me3 coupled to mass spectrometry, in order to identify regulators of enhancer-promoter communication. Apart from CTCF, equally essential was YY1 which was highly expressed in the majority of the examined cell types. Moreover, YY1 was found to mediate chromatin interactions between promoter and enhancer regions, whereas CTCF was mainly found in insulator regions. It was also previously shown that YY1’s interplay with RNAs was important for its stabilization on DNA (43). Similar to CTCF, YY1 forms homodimers and this feature is highly dependent on its interplay with various RNA molecules. RNA-seq experiments identified equal down- as well as up-regulated gene expression in the absence of YY1, highlighting once more its bipartite role in transcriptional regulation.

Loss of YY1 leads to major loss of enhancer-promoter communication for key developmental genes, correlated to their downregulated expression, while ectopic expression of YY1 rescues these defects (44). The latter study highlighted the layers of gene expression regulation in the 3D genome of mESCs, as CTCF is responsible for the formation of a general high-order of TAD structures, whereas YY1 acts in subdomains of TADs to regulate enhancer-promoter communication.

## SATB1 Shapes Thymocyte Development by Controlling 3D Genomic Organization

As previously mentioned, global genome organizers seem to play crucial roles in mediating transcriptional regulation of major T-cell-specific genes, but many studies also point out the necessity of tissue-specific regulators. Depletion of either CTCF or Cohesin complex subunits impacts the gene expression pattern but not to a great extent, indicating that along with these general chromatin organizers there are multiple factors expediting tissue-specific and spatiotemporal enhancer-promoter communications in governing the tissue-specific expression programs (149).The role of global genome organizers is of crucial importance in mediating transcriptional regulation through 3D genome interactions, but when assessing a vast array of cases, their function is synergistic and depends on tissue specific factors in order to control tissue specific transcriptional regulation.

The earliest record of special AT-rich sequence-binding protein-1 (SATB1) in the literature was by Dickinson et al.,1992 (150), where SATB1 was described to bind the nuclear matrix-associated DNA (MAR/SAR regions) in the nucleus. This publication also provided the first indications that this protein is a cell-specific nuclear factor, binding to double-stranded DNA *via* recognition of AT-rich sequences. However, since then, other studies have uncovered that SATB1 is mainly expressed in the thymus, the bone marrow, the brain and in cancer cells. In multiple cell types (ESCs, T and B cells), SATB1 regulates gene expression by folding chromatin into loop domains, tethering specialized DNA elements to its network. Several studies have uncovered that ablation of SATB1 by gene targeting, resulted in temporal and spatial misexpression of numerous genes and arrested T-cell development, supporting its role as a cell-type specific genome regulator (151–153). SATB1 is a transcription factor with a capacity to multimerize and has multiple DNA binding domains, specifically CUT1, CUT2 and a C-terminal homeodomain (HD). The CUT1 domain is the most indispensable for efficient chromatin binding and can single-handedly confer the high affinity binding attributed to SATB1. The HD implements binding specificity *via* distinct characteristics on the DNA, namely negative propeller twist (PT) and higher AT DNA content than the genome average (154). SATB1’s protein domains facilitate efficient DNA binding and maintain its functional and structural resilience to torsional stress which is caused by eu- and hetero-chromatinization in the nucleus. Transcription factors (TFs) like SATB1 are characterized by their rapid dissociation from their binding sites in order to either attach to an alternative locus or form complexes to exert their functions in response to external (TCR, NF-kB signalling) or internal (reactive oxygen species (ROS), metabolic pathways) stimuli (151–153).

SATB1 forms cage-like structures in the T cell nucleus with a distribution mainly in euchromatic regions. On a few occasions, SATB1 has also been detected in heterochromatic regions, though not as densely as in the euchromatic regions mentioned above (155). This signifies that, depending on the cell type and developmental stage assessed, SATB1 is a highly motile and versatile molecule distributing itself on different genomic loci, while also associating with multiple complexes (ACF, ISWI, and HDAC1) in response to a stimulus. These complexes are known to cause de-acetylation at SATB1-specific bound sites (156). Studies performed on SATB1 conformation and structure showed that it is phosphorylated by protein kinase C (PKC), which binds to its PDZ-like domain. This posttranslational modification of SATB1 is the quintessential characteristic that facilitates signalling and recruitment of protein complexes at genomic loci. More specifically, this modification dictates binding to specific protein complexes (HDAC1 or PCAF) at SATB1’s bound loci. The preference of complex binding, depending on phosphorylation, determines repression (phosphorylated SATB1-HDAC1 interaction in resting cells) or de-repression (dephosphorylated SATB1-PCAF interaction in activated cells) of target genes, like IL-2. A similar model can be considered for all genes regulated by SATB1 (157). Other important remodeling complexes, like NURD, also interact with SATB1, with the latter acting as a “docking platform” for the complex (151, 157, 158). The alternate interplay of these complexes bound to SATB1 results in disparate effects. Recruitment of the NURD complex to the IL2Ra locus causes its de-acetylation, resulting in gene repression, hence giving rise to the defective effector (Teff) and regulatory (Treg) T cell development (151, 157). Given its important properties in regulating gene expression, SATB1 is essential in determining cell development in both the thymus and the peripheral lymphoid organs. This characteristic makes it one of the most well-studied transcription factors for both thymocytes and immune cells in general.

Although it was previously shown to bind on AT-rich regions of the genome, SATB1 protein associates with different areas of the genome of varying characteristics, determining gene expression in multiple cell types and processes. This variance in SATB1 implication for regulating cell processes spans from epidermal differentiation, breast cancer metastasis, thymocyte development, Th2 cell activation and cytokine production, cortical development, X-chromosome inactivation, and embryonic stem cell differentiation (154).

One of the most important functions of SATB1 is regulating thymocyte development. Studies performed have pinpointed the highest levels of expression for this protein, hence hinting at its importance in regulating gene expression and fine-tuning different developmental stages in the thymus. The double negative (DN) thymocyte population is characterized by minimal SATB1 expression, in contrast, the double-positive (DP) population exhibits the highest level of expression, mimicking expression at the DN4 stage of development. Furthermore, when analyzing both CD4^+^ and CD8^+^ single-positive (SP) cells, SATB1 expression is considered moderate and fluctuating. More specifically the CD4^+^CD8Lo intermediate thymocyte population evinced a rather higher expression of SATB1 in comparison to the DP subset. In general, CD4^+^ SP thymocytes demonstrate the bimodal distribution of SATB1 expression resulting in two distinct CD4^+^ T cell subpopulations (SATB1Hi and SATB1Lo). Interestingly, assessment of the CD8^+^ T cell population indicated a SATB1 expression profile mimicking that of the CD4^+^ SATB1Lo T cell subset. Finally, although there is downregulated SATB1 expression in mature CD4^+^SP thymocytes, the expression is re-established in response to TCR signalling in CD4^+^ cells in the secondary lymphoid organs. This was reported to occur in response to signals like cytokines and antigenic TCR stimulation (159).

When determining thymocyte cell fate and development, multiple studies have inextricably linked SATB1 to lineage selection and preference in response to external stimuli. To assess SATB1’s function in different stages of thymocyte development, several conditional knockout (complete knockout is post-embryonic lethal) murine models have been developed, deleting *Satb1* at discrete developmental stages. Such models include *Satb1* ^f/f^ *Vav-Cre* (haemopoietic stage), *Satb1* ^f/f^ *Lck-Cre* (early CD4^+^ CD8^+^ double-negative stage) and *Satb1* ^f/f^ *CD4-Cre* (transitional CD4^+^ CD8^+^ double-positive stage).These different models have provided critical insights into the molecular mechanisms governing T cell development and lineage specification and established *Satb1’s* role as an important chromatin regulator. Researchers have identified four alternative *Satb1* gene promoters (P1, P2, P3, and P4). Their interchanging usage determines the alternate *Satb1* transcript expression observed. The preference of promoter usage in *Satb1* gene expression is ever-changing depending on the developmental stage and relies on TCR signaling. In DP thymocytes, P1, P3, and P4 transcript variants are the most abundant with the P3 transcript variant being the most integral amongst the *Satb1* transcript variants. In CD4SP thymocytes, however, the P2 transcript variant is highly expressed along with the P3 transcript. The same expression pattern is not present in CD8SP thymocytes (160). *Satb1* is regulated by TCF1, a transcription regulator that controls the expression of T cell lineage specification transcription factors, including ThPOK, RUNX3, and the T-reg specific factor FOXP3. In CD4^+^ T cell lineage, SATB1 was shown to be involved in *Cd4* activation, by regulating the activation of enhancers in the *Cd4* locus (161). In this regard, a recent study showed that a proximal enhancer (E4p) of the *Cd4* gene is required for stage-specific DNA demethylation of *Cd4* intronic regions, which then contributes to establishing stable *Cd4* expression in mature T cells (162). SATB1 controls CD4 cell lineage specification by controlling the looping of different enhancers on the *Cd4* gene promoter (161).

SATB1 also binds to nucleosome-dense regions and studies have gone as far as characterizing it as a pioneer factor in immune cells, like regulatory T cells (T-regs) (163). Pioneer factors regulate chromatin accessibility and define early developmental stages in lineage specification. This publication supports this notion by establishing SATB1 as an indispensable factor in regulating the association of Treg super-enhancers (T-reg-SEs) with key T-reg cell signature genes, long before the expression of FOXP3, a fundamental transcription factor for thymic Treg (tT-reg) cell development and differentiation. As a result, the timing of *Satb1* deletion determines its effect on Treg cell development. Its ablation in thymocytes prior to *Foxp3* expression predominantly impairs tT-reg cell development, whereas its deficiency in mature conventional T (T-conv) cells seems to promote peripheral Treg (pT-reg) cell differentiation. Studies have also revealed the opposing role of the two factors, as FOXP3 binds in the genomic region of *Satb1* and represses its expression in Tregs, whereas ectopic expression of SATB1 converts Tregs to conventional T cells. More specifically, *Satb1* deficiency before, but not after, the FOXP3^+^ thymic Treg stage impairs Treg-SE activation and, consequently, the expression of Treg cell signature genes, leading to a severe autoimmune phenotype in mice (163–165).

Evidently, SATB1 was shown to be involved in both chromatin organization and positive selection of T cells and its loss of expression leads to an autoimmune phenotype. This underlines the role of this factor in mediating gene regulatory networks in developing and mature T cells, which then facilitates the establishment of immune tolerance in mice. Over-activation of some cell types (Treg cells) and the underrepresentation of others (CD8^+^SP) skew the fine balance of the immune response in mice, towards a pro-inflammatory and self-reactive phenotype (165). All this research performed throughout the years has established SATB1 as an essential genome organizer that is indispensable in nearly all stages of T cell development, in both humans and mice.

## SATB1 in (Patho)Physiology

During the early attempts to understand the mechanisms of (patho)physiological conditions leading to the onset of disorders like cancer, it was uncovered that multiple proteins are implicated in this process. The BUR-binding proteins like poly (ADP-ribose) polymerase (PARP-1) and p53 were isolated from cancer cells and their overexpression was observed, among others, in aggressive human breast cancer cells, linking their levels of expression to the tumorigenic potential of normal cells (166). Such a BUR-binding protein is SATB1.

SATB1 was found to be expressed in aggressive breast cancer cells, though firstly not present in human mammary epithelial cells. Research into this also showed that SATB1 was rather only expressed in some carcinoma cells and in some patients and not in all cell types from the tissues examined, hence providing insight into the heterogeneity of tumor progression and metastasis potential. More specifically, the presence of SATB1 was known to interact with and upregulate genes responsible for poor prognosis in breast cancer. Some processes affecting metastasis that were affected by SATB1 expression are the following: cell adhesion, signalling, extracellular matrix (ECM) formation, and the cell cycle. Factors upregulated by the presence of SATB1 affecting metastasis potential are: vascular endothelial growth factor B (VEGFB), matrix metalloproteinases (MMP2, 3, and 9), transforming growth factor-B1 (TGFB1), and connective tissue growth factor (CTGF) (155). SATB1 was also associated with a worse prognosis in cancers, like laryngeal squamous cell carcinoma (LSCC)(in mucosal cells) (167), endometrioid endometrial cancer (EEC) (in EEC cells) (168), hepatocellular carcinoma (HCC)(in human hepatocarcinoma tissue and liver cancer cell lines) (169), rectal cancer (in human rectal cancer tissues) (170), cutaneous malignant melanoma (CMM)(in primary CMM cells) (171), gastric cancer (in gastric cancer cells) (172) and prostate cancer (173). On the contrary, SATB1 overexpression leads to a better survival rate in non-small cell lung carcinoma patients (174). This underlines the duality of SATB1 function in cancer depending on the cell type in which it is expressed (selective expression in tumor cells and not in the surrounding tissues).

**Figure 2:**
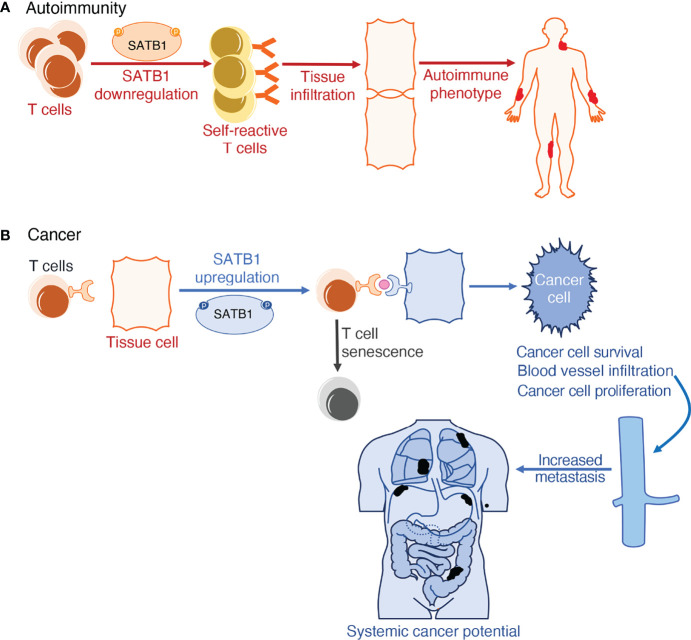
Schematic representation of SATB1 deregulation in (patho)physiology. **(A)** SATB1 downregulation in thymic T cells results in defective T cell training in the thymic medulla. When these self-reactive T cells circulate in the lymphatic system, they fail to recognize pathogens and instead misidentify a person’s autoantigens as hostile. This induces T cell infiltration in tissues, further accentuating inflammation observed in autoimmune phenotypes in both humans and mice. **(B)** SATB1 expression upregulation in normally non-expressing tissue cells is the tell-tale mark of multiple cancers. SATB1 boosts the expression of the PD1/PDL1 pathway which is responsible for both T cell senescence and exacerbating deterioration markers in cancer; namely cancer cell proliferation, differentiation, survival, and potential for metastasis.

When investigating the molecular mechanisms at work in determining the plurality of cancers and their outcomes, a link with long-range chromosome looping was uncovered. Proteins involved in higher-order interphase chromosome structure formation, like CTCF and SATB1, have inextricably been linked to the deregulation of gene expression. By forming long-range loops, they bring to proximity genes that may not be meant to interact with each other, thus interfering with the proper regulation of their expression. This deregulation in gene expression can contribute to the multiplicity of phenotypes observed in cancers (175–177). Apart from abnormal gene expression, mediated by high order looping, some cancer cells evade immune detection and clearance by upregulating the expression of the Programmed Cell Death Protein 1 and its ligand (PD1-PDL1) pathway. PD1 has an essential role in hindering immune responses and determining self-tolerance *via* controlling T-cell activity, promoting apoptosis in antigen-specific T cells, and impeding regulatory T cell apoptosis. The PD-L1 protein can bind to PD1 and cause contraction of the proliferation of PD-1 positive cells, inhibit their cytokine secretion, and induce apoptosis. It is also linked to attenuation of the immune response to tumor cells. By upregulating this interaction, cancer cells mimic normal macrophages, some activated T and B cells, dendritic cells and some epithelial cells, where these molecules are expressed, particularly under inflammatory conditions, in order to downregulate anti-tumor responses from T cells (178). Studies have uncovered that SATB1 also plays a role in inducing histone deacetylation at genomic regions regulating PD-1 expression, hence limiting PD-1 expression on the cell surface. As a result, SATB1-deficient T cells failed to repress the elevation of inhibitory PD-1 upon TCR activation, limiting the function of tumor-reacting T cells. This culminates to diminished responsiveness of T cells *in vivo* in the tumor cells expressing PD-L1 (**Figure 2**) (158).

SATB1 was also implicated in several cutaneous T cell lymphoma syndromes development. Downregulation of its expression led to abnormal upregulation in the NOTCH1/STAT5/CCR4 pathway in the bone marrow and CD4^+^ cells found in the secondary lymphoid organs of Sézary syndrome patients (179). The same downregulation in expression was also observed in mycosis fungoides syndrome T cells, where the proto-oncogenic JAK3/STAT5/miR155 pathway was activated upon lack of SATB1 expression (180).

When assessing SATB1’s function in autoimmune disorders, a good model of investigation is the induction of experimental autoimmune encephalitis (EAE) in mice. Using *Satb1* ^f/f^ *Vav-Cre* and *Satb1* ^f/f^ *Lck-Cre* mice, it was observed that T cells derived from these mice fail to differentiate into pathogenic T cells during the priming or induction phases of EAE. This finding supports the notion that in EAE, *Satb1* overexpression has a deteriorating role by promoting pathogenic Th17 cell generation and effector function (181). Furthermore, SATB1 is also important for the onset of the autoimmune Sjögren’s syndrome in mice. As this factor is indispensable for the development of tTreg cells, but not for the differentiation of pTreg cells, the majority of Treg cells in the secondary lymphoid organs of *Satb1*conditional knockout mice are pTreg cells. Studies have shown that all Treg cells in those mice had a defective phenotype and hence were unable to downregulate abnormal pathogenic T cell activation and tissue infiltration, resulting in the development of autoimmune phenotypes (**Figure 2**) (182).

## Discussion

The foundation of adaptive immunity is laid upon the development of T cells in the thymus during the early embryonic stages, for both humans and mice. Mammalian genomes are characterized by their higher order of chromatin structure in the 3D nuclear space, which is crucial for gene expression and the epigenetic regulation governing thymic development. The differentiation of several T cell lineages depends on multiple global and tissue specific transcription factors (histone modifiers and chromatin remodelers) controlling spatiotemporal gene expression (183–185). These proteins in association with cis-regulatory regions (enhancer and promoter elements) along the genome, define the dynamic conformation of chromatin. Previous work has identified the important role of global chromatin organizers such as CTCF and YY1 in gene regulation and expression. When considering T cell development though, SATB1, as a tissue-specific chromatin organizer, is purportedly playing a crucial role in the developmental landscape patterning alongside the aforementioned global organizers (**Table 1**). SATB1’s posttranslational modifications regulate its association with DNA and subsequently determine the epigenetic profile of several genes. SATB1 has been shown to interact and recruit many key players necessary for gene expression regulation, but the mechanism by which SATB1 regulates the promoter-enhancer communication in T cells remains elusive. The role of SATB1 in T cell specific gene regulation has been studied using various conditional knockout models. The absence of SATB1 leads to arrested T cell development at the DP stage and apoptosis accompanied by increased levels of effector CD4 T cells in the periphery, distinctive features of autoimmune-like diseases. Additionally, loss of SATB1 from the DN4 stage skews T cell development towards immature, non-activated Tregs, whereas T_conv_ development is hindered. SATB1 was also found to play a profound role in the pre-patterning of the T_reg_ super enhancer landscape at earlier developmental stages. Blockade of SATB1 at the DP stage promotes the underrepresentation of CD8SP T cell subsets. Deregulation in SATB1 expression has been observed in multiple T cell derived autoimmune diseases. As such, ectopic expression of this factor in several cell types and tissues correlates with worse prognosis in multiple cancers. Further investigation is required in order to identify how SATB1 regulates the T cell chromatin loopscape structure. This will prove essential in the context of its interplay with major transcription factors when attempting to elucidate the role of genome architecture in epigenetic regulation. Uncovering this mechanism will shine a light on the meandering path towards solving severe pathological conditions in which SATB1 can act as a therapeutic target.

**Table 1:** Mouse models of genome organizers and their respective phenotype affecting T cell physiology.

Mouse model	Phenotype	Reference
*Satb1^-/-^*	- mice are small in size, small thymi and spleens, die at 3 weeks of age - reduced numbers of immature CD3^−^CD4^−^CD8^−^ triple negative thymocytes - thymocyte development blocked at the DP stage - peripheral CD4^+^ undergo apoptosis and fail to proliferate in response to activating stimuli	(152)
*Satb1* ^fl/fl^ *Cd4*-Cre^+^	- Impaired Treg super enhancer activation - Impaired expression of T_reg_ signature genes - Severe autoimmunity due to T_reg_ cell deficiency - IgE hyperproduction - reduced efficiency of thymocyte differentiation into CD4SP thymocytes - inappropriate T cell lineage specification after MHC class I/II-mediated selection - failure to generate NKT and T_reg_ cells	(161, 163)
*Satb1* ^fl/fl^ *Vav*-Cre^+^	- deficient positive selection at the CD4^+^CD8^+^ double-positive (DP) stage during T cell development in the thymus - reduction in SP thymocytes - development of autoimmune disease - reduced numbers of Foxp3^+^ regulatory T (T_reg_) cells - impaired suppressive function of T_reg_ cells - impaired negative selection during T cell development in the thymus - deregulated *Rag* expression	(165, 186)
*Satb1* ^fl/fl^ *Lck*-Cre^+^	- autoimmunity	(165)
*Ctcf1* ^fl/fl^ *Lck*-Cre^+^	- defective TCRαβ lineage development - accumulation of late double-negative and immature single-positive cells in the thymus - cell cycle arrest during TCRαβ lineage development	(125)
*Yy1* ^fl/fl^ *Lck*-Cre^+^	- higher percentage of DN thymocytes - significantly reduced number of DP thymocytes - reduced viability of DP thymocytes due to increased apoptosis	(187)
*Yy1* ^fl/fl^CD2-Cre^+^	- significantly reduced TCRβ-expressing thymocytes - substantially increased percentage of γδ T cells - increased percentage of DN3 (CD25^+^CD44^−^) thymocytes - reduced percentage of DN4 (CD25^−^CD44^−^) thymocytes	(187)
*Yy1* ^fl/fl^ *Cd4*-Cre^+^	- blocked *i*NKT cell development - impaired thymocyte survival - deregulation of *Plzf* gene expression	(188)

DP, double positive; DN, double negative; SP, single positive; YY1, Yin-Yan 1; IgE, immunoglobulin E; TCR, T cell receptor.

## Author Contributions

GP and D-AP wrote the first draft of the manuscript. CS edited the manuscript and figures. All authors contributed to the article and approved the submitted version.

## Funding

GP was supported by a fellowship from aDDRess-H2020-MSCA-ITN (GA812829). D-AP was supported by the Hellenic Foundation for Research and Innovation (HFRI) under the 3rd Call for HFRI PhD Fellowships (Fellowship Number: 5707).

## Conflict of Interest

The authors declare that the research was conducted in the absence of any commercial or financial relationships that could be construed as a potential conflict of interest.

## Publisher’s Note

All claims expressed in this article are solely those of the authors and do not necessarily represent those of their affiliated organizations, or those of the publisher, the editors and the reviewers. Any product that may be evaluated in this article, or claim that may be made by its manufacturer, is not guaranteed or endorsed by the publisher.
